# Ultrasonographic Findings in 41 Dogs Treated with Bone Marrow Aspirate Concentrate and Platelet-Rich Plasma for a Supraspinatus Tendinopathy: A Retrospective Study

**DOI:** 10.3389/fvets.2018.00098

**Published:** 2018-05-17

**Authors:** Renee A. McDougall, Sherman O. Canapp, Debra A. Canapp

**Affiliations:** Veterinary Orthopedic and Sports Medicine Group, Annapolis Junction, MD, United States

**Keywords:** supraspinatus tendinopathy, mesenchymal stem cells, bone marrow aspirate concentrate, platelet-rich plasma, regenerative medicine, musculoskeletal ultrasound

## Abstract

**Objective:**

To report sonographic findings for dogs with a supraspinatus tendinopathy (ST) treated with an ultrasound-guided intratendinous injection of bone marrow aspirate concentrate (BMAC) and platelet-rich plasma (PRP).

**Methods:**

Medical records for dogs diagnosed with an ST and treated with a BMAC-PRP injection were reviewed. Data collected included patient signalment, radiographic findings at the time of initial evaluation, and sonographic findings, including cross-sectional area (CSA), fiber pattern, and echogenicity.

**Results:**

Of 70 records reviewed, 41 met the inclusion criteria. Mean CSA of the supraspinatus tendon decreased by 0.06 cm^2^ between baseline and 45 days post-treatment (*p* = 0.0025), and 0.09 cm^2^ between baseline and 90 days post-treatment (*p* < 0.0001). Analysis of CSA in dogs with a unilateral ST at baseline revealed a difference of 0.08 cm^2^ between the affected and unaffected tendon at baseline, with the affected tendon measuring larger than the contralateral tendon (*p* < 0.0001). This difference became statistically insignificant by 45 days after treatment (u_1_-u_0_ = 0.04 cm^2^, *p* = 0.2855) and remained so 90 days post-treatment (u_1_-u_0_ = 0.03 cm^2^, *p* = 0.1910). In most cases (90.6%), the fiber pattern and echogenicity was considered improved 90 days post treatment. In a minority of these cases (13.8%) the fiber pattern and echogenicity abnormalities were considered resolved.

**Conclusions:**

Using qualitative and quantitative sonographic measures, BMAC-PRP was associated with an improvement in supraspinatus tendon size, fiber pattern, and echogenicity. Given the protracted nature of tendon healing, long-term evaluation may reveal continued improvements in chronic structural changes not captured during the current study. Functional studies are required to evaluate the clinical benefits of BMAC-PRP in the treatment of STs in dogs.

**Clinical significance:**

An ST is a common contributor to forelimb lameness in dogs and remains notoriously difficult to treat. Previous studies have been associated with inconsistent treatment outcomes. In the current study, BMAC-PRP is investigated as a minimally invasive treatment option, revealing positive sonographic results.

## 1. Introduction

Forelimb lameness in dogs is often multifactorial in nature. One component commonly identified in those with chronic mild to moderate lameness is supraspinatus tendinopathy (ST).

Along with other tendinopathies, a diagnosis of an ST may be confirmed using MRI or musculoskeletal ultrasonography (1–9). Both have been validated for the diagnosis of STs (2, 4, 8), however, cost, access to advanced imaging technology, and the requirement for general anesthesia often limits the clinical applicability of MRI. In contrast, diagnostic ultrasound allows for increased ease of acquisition without the need for sedation or anesthesia, and the ability to make contralateral comparisons. An ability to evaluate the contralateral limb may be particularly valuable in dogs with bilateral forelimb lameness, reported in up to 37.6% of dogs with a supraspinatus tendinopathy (10), making diagnostic ultrasound a convenient modality for both patient diagnosis and an evaluation of patient response to treatment over time.

Sonographic findings consistent with pathology of the supraspinatus tendon may include changes in fiber pattern, echogenicity, and cross-sectional area (CSA) (1). 

Fiber pattern and echogenicity of the supraspinatus tendon have been previously evaluated in both humans and dogs, along with well-established markers of tendinopathy including a heterogenous fiber pattern, indicative of intratendinous hemorrhage or an acute partial tear, regions of hypoechogenicity, consistent with intratendinous edema, and regions of hyperechogenicity or acoustic shadowing, evidence of scar tissue formation or intratendinous calcification (4, 11–15). These markers are often used for diagnostic purposes, (2, 15–17) however, changes in fiber pattern and echogenicity have also been used to evaluate patient responses to treatment (16, 17). 

In addition to fiber pattern and echogenicity, CSA is also considered a valuable and objective finding commonly obtained during tendon sonography. Increases in tendon thickness or CSA have been previously validated as markers of tendinopathy in both humans and horses, and are considered reliable and repeatable components of sonographic tendon analysis (18–21). Changes in tendon CSA or thickness have also been previously used in dogs to diagnose STs and evaluate the response of the supraspinatus tendon to treatment (10, 16, 17).

STs are notoriously difficult to treat. In one previous retrospective study evaluating 327 dogs diagnosed with an ST, 75% had failed to respond to non-steroidal anti-inflammatory drugs (NSAIDs), and 40% had failed a dedicated rehabilitation program (10). Smaller case series investigating outcomes associated with treatment of STs in dogs have reported improvement in patients treated using formal rehabilitation, including extracorporeal shockwave ultrasound, and open surgery, however, resolution remains elusive in some cases, with persistence or recurrence of lameness noted in up to half of all surgically treated, and 33% of non-surgically treated, cases (15, 22, 23).

Intratendinous injection of mesenchymal stem cells (MSC), represents an alternative treatment that has demonstrated promising clinical benefits (16, 24–27). The potential advantages of using such cells include their regenerative capacity, or their ability to differentiate into tenocytes, as well as their ability to modulate the local inflammatory response, regulate cellular apoptosis, and induce neovascularization (24, 28–32).

As a treatment modality, the administration of stem cells is often coupled with platelet-rich plasma (PRP), which provides the fibrin scaffold required for MSC survival, as well as growth factors that enhance endurance and assist with stem cell activation and differentiation (33–36).

Many *in vitro* and laboratory animal studies exist investigating the use of MSC to treat STs, however, to the author’s knowledge, there is only one study has been published describing the use of MSC and PRP in a veterinary client-owned population (16). This study demonstrated both a clinical and ultrasonographic improvement in dogs with an ST that were treated using adipose-derived progenitor cells (ADPC), a culture-expanded MSC population and PRP.

Though promising, further investigation into ADPC and PRP (ADPC-PRP) as a treatment for STs has largely been discontinued due to publication of new FDA guidelines that discourage the use of autologous type I animal stem cell products, which include those that have been manipulated by culture expansion (37).

To this end, additional, minimally manipulated, sources of MSCs have been sought, including bone marrow-derived stem cells such as those found in bone marrow aspirate concentrate (BMAC), a type II autologous animal stem cell product (37).

Additional advantages include the ability to prepare and deliver the bone marrow-derived stem cells at the time of diagnosis. This point-of-care delivery system allows for reduced client costs and the administration of regenerative treatments without the need for an additional anesthetic event. BMAC may also be collected with increased ease and decreased patient morbidity when compared with ADPC, which often involves intra-abdominal penetration.

Therefore, further investigation into BMAC as an alternative MSC treatment for STs in dogs is essential. The purpose of this retrospective study was to evaluate the effects of intratendinous BMAC-PRP on sonographic findings in dogs diagnosed with an ST.

## 2. Materials and Methods

### 2.1. Medical Records Review

In accordance with AAALAC International Rules of Accreditation, this study was performed with the approval of the VOSM Research Committee and with owner consent. All clients provided written consent as required by Veterinary Orthopedic and Sports Medicine Group for every study participant. Medical records were reviewed for dogs diagnosed with an ST that were treated with an ultrasound-guided intratendinous injection of BMAC-PRP. Baseline patient data collected included signalment at the time of treatment, status of the patient as a working or companion dog, duration of lameness prior to initial presentation, limb involvement, and orthopedic physical exam findings related to the diagnosis of an ST, including pain on shoulder manipulation and direct palpation of the supraspinatus. The presence of shoulder radiographs, and radiographic changes consistent with an ST, including mineralization or sclerosis in the region of the supraspinatus tendon, were recorded.

#### 2.1.1. Treatment

The date of treatment was recorded. BMAC and PRP generated according to the protocols described below.

One hind limb was chosen for bone marrow collection. Bone marrow aspirate (BMA) was then harvested using previously described techniques (38, 39). For all dogs a total of 30–40 ml aspirate was drawn into a 60 ml syringe containing 5 ml of heparin. The BMA was then processed according to the manufacturer to create BMAC.^1^ The Jamshidi needle was removed from the 60 ml syringe and the BMA was transferred into the Concentrating Device (CD) provided. A counterbalance was created for the CD based on the amount of BMA collected, and both the CD and counterbalance were placed in a centrifuge for 10 min at 3,600 rpm.^2^ The initial centrifugation resulted in a layer of plasma atop stem cell concentrate. Therefore a second 60 ml syringe was then attached to the CD and aspiration of the light pink plasma layer was performed until red was seen entering the connecting tube between the CD and syringe. A 10 ml syringe was then attached to the CD to aspirate the remaining contents of the CD, the BMAC. The resulting volume was 10% of the original volume, and therefore commonly 3–4 ml. In humans, this solution has been previously proven to contain a total nucleated cell count 6.5 times that of BMA, a CD34+ hematopoietic stem cell concentration 11.5 times that of BMA, and a reduced hematocrit of 10% or less (40). The solution is currently in the process of validation in canines, however preliminary data indicates a 50% nucleated cell count, with 94–99% viability (41). Adherence to a plastic surface was demonstrated using a colony forming unit assay (CFU), revealing a rate of 26% colonies formed per CFU (41), suggesting the presence of MSCs (42). Demonstration of multipotency and expression of species-specific stem cell surface markers, are currently under way.

According to manufacturer instructions, the blood volume required for a standard volume of PRP (4 ml) was obtained using the following methods. One jugular vein was chosen for PRP collection. The selected jugular vein was clipped and aseptically prepared. Approximately 50 ml of whole blood was collected using an 18-gauge butterfly needle and 60 ml syringe containing 10 ml CPDA anti-coagulant. The whole blood was then processed according to the manufacturer.^3^ The syringe containing whole blood was transferred to the CD provided. A counterbalance was created for the CD based on the amount of whole blood collected. Both the CD and the counterbalance were placed in a centrifuge for 1 min at 3600 RPM.^2^ The resultant suspension was a platelet rich buffy coat and platelet poor supernatant atop red blood cells. A 60 ml syringe was used to aspirate the platelet rich and poor layer, and transfer them to a second 30 ml CD. An appropriate counterbalance was again created, and the CD and counterbalance were placed in a centrifuge for 5 min at 3800 RPM. The final suspension consisted of a platelet poor supernatant above a platelet rich buffy coat. A 30 ml syringe was attached to the 30 ml CD and all but 4 ml of plasma aspirated. A 10 ml syringe was then attached to the CD and the remaining plasma gently mixed. The remaining 4 ml of PurePRP was drawn into the 10 ml syringe. The final solution has been previously validated as a product with a 6-fold increase in platelet count, as well as an increase in monocyte and lymphocyte concentrations, and a decrease in red blood cell and neutrophil concentrations (43). Though canine guidelines do not yet exist, this product meets human PRP guidelines, which recommend a 4–7 fold increase in platelet concentration (44–47). In comparison with other commercial canine PRP systems, the PRP system used in the current study has been reported to deliver the greatest platelet yield (43). This system is similar to most canine PRP systems in the reduction of red blood cells and neutrophils, both of which have been associated with the presence of deleterious inflammatory cytokines that may lead to synovial cell death as well as the degradation of collagen and other components of the joint capsule extra-cellular matrix. The concentration of both lymphocytes and monocytes were increased in this product, though the effect of these white blood cells in PRP remain unknown.

The BMAC was administered using a previously described fenestration technique: (48, 49) A 22-gauge 1.5″ needle was inserted along the long axis of the tendon and parallel to the ultrasound transducer. Once the needle was identified in the ultrasound field, the needle was advanced toward the lesion and the BMAC was administered under ultrasound guidance. The PRP was similarly administered by replacing the syringe containing BMAC with the syringe containing PRP while maintaining the intra-tendinous location of the needle. The volume delivered was as close to a 1:1 ratio of BMAC-PRP as possible. The exact volume delivered was determined by the administering clinician and discontinued when disruption of supraspinatus fascicles became visible sonographically.

#### 2.1.2. Outcome

Ultrasounds were performed by a single author (DC). Ultrasound data was collected at baseline, 45, and 90 days after BMAC-PRP treatment as is routinely recommended at the time of treatment. Due to variation in patient follow-up, the closest patient re-evaluation to the recommended follow-up dates were selected for data abstraction. For patients with a second date of evaluation, any evaluation following initial discharge and prior to 90 days post-treatment was eligible for selection. For patient with a third date of evaluation, any evaluation following that selected for inclusion as “45 days post-treatment,” and more than 45 days post-treatment was eligible for selection. Diagnostic musculoskeletal images were collected bilaterally using an 18 MHz linear probe.^4^ Both shoulders were clipped and cleaned. Patients were then placed in lateral recumbency. Ultrasonographic images were obtained in a transverse and longitudinal orientation.

Collected data included CSA, abstracted for at all time points. In addition, both general trends and specific fiber pattern and echogenicity abnormalities consistent with an ST were abstracted from ultrasound reports at baseline and 90 days after treatment.

##### 2.1.2.1. CSA

Cross-sectional area was measured in the transverse plane. Three measurements were obtained per tendon. The final CSA was an average of these measurements.

To evaluate how the size of the affected tendon changed over time, the difference in CSA between 45 days post-treatment and baseline, and 90 days post-treatment and baseline, was determined.

To evaluate how the size of the affected tendon changed as it compared to the size of a normal supraspinatus tendon, the difference in CSA between the primary and contralateral limb was determined at baseline and 90 days post-treatment. This calculation was performed only for those dogs that were diagnosed with a unilateral ST.

##### 2.1.2.2. Fiber Pattern and Echogenicity

Information regarding tendon fiber pattern and echogenicity in the primary limb was assessed, including the presence of a generalized heterogenous or mottled fiber pattern, a generalized hypoechoic fiber pattern, foci of hypoechogenicity or hyperechogenicity, evidence of calcification, and evidence of contact and/or impingement of the biceps tendon. Findings identified 90 days post-treatment were compared with those noted at baseline by a single author (RM). Fiber pattern and echogenicity were considered improved if the sonographer noted improvement within the ultrasound report, or if a comparison of the reports revealed improvement in one or more of the above categories. Findings were considered unchanged if the sonographer noted a static appearance to the supraspinatus tendon, or if a comparison revealed no change in any of the categories above. An outcome of deterioration was assigned if either fiber pattern or echogenicity had worsened as determined by the sonographer or the author reviewing the reports.

#### 2.1.3. Exclusions

Patients were excluded if quantitative comparisons for CSA could not be made, as this was the primary quantitative outcome measure for this study. For patients with bilateral forelimb lameness, the more affected limb was considered primary. For unilateral analyses, patients with bilateral forelimb lameness or a diagnosis of bilateral STs, were excluded.

#### 2.1.4. Statistics

Distributions of continuous data were assessed for normality by the Shapiro-Wilk test. Comparisons of paired, normally distributed, data were made with the paired Student’s *t*-test. Proportional distributions of categorical data were compared with the chi-square test or Fisher’s exact test. A *p*-value of <0.05 was applied. A post-hoc power analysis revealed a power of 51% given a sample size of 41 dogs.

## 3. Results

### 3.1. Inclusion

70 dogs met the inclusion criteria for medial record review. Of these, 29 records did not contain enough information to assess change in CSA over time and were excluded. The remaining 41 dogs were included in the final statistical analysis.

### 3.2. Medical Record Review

The mean age of the final cohort was 5.5 ± 2.6 years (range 0.8–11.1 years). Seventeen dogs were female (two intact), and 24 dogs were male (10 intact). The most common breed was the Labrador Retriever 11, followed by mixed breed dogs 5, Border Collies 3, Doberman Pinschers 3, Chesapeake Bay Retrievers 3, Golden Retrievers 2, German Shepard Dogs 2, Rottweilers 2, Standard Poodle 2, and one of each of the following: Bernese Mountain Dog, Polish Sheepdog, Malamute, Coonhound, Welsh Corgi, American Staffordshire, Samoyed, and English Labrador Retriever. Among those with a listed occupation, slightly more than half were companion dogs (*N* = 20, 51.3%), with the remainder being working or competitive dogs (*N* = 19, 48.7%).

The median duration of lameness prior to presentation was 9 (range 1–72) months.

A supraspinatus tendinopathy was diagnosed bilaterally in over half of all dogs (*N* = 26/41, 63.4%), and unilaterally in the remainder (*N* = 15/41, 36.6%).

Findings at the time of initial evaluation included pain on manipulation of the affected shoulder, including flexion or extension (29/31, 93.5%) and direct palpation of the supraspinatus (27/31, 87.1%).

Bilateral shoulder radiographs were obtained in 32/41 (78.0%) dogs. Mineralization in the region of the supraspinatus tendon was noted in 12/32 cases (37.5%). Mineralization was unilateral in 6/12 cases (5 of which had been diagnosed with bilateral STs), and bilateral in the remaining 6/12 (5 of which had been diagnosed with bilateral STs).

### 3.3. Outcome

On average, the post-treatment evaluation closest to 45 days after injection was 46 ± 11 days after injection. Similarly, the average post-treatment evaluation closest to 90 days following treatment was 96 ± 14 days after injection.

#### 3.3.1. Musculoskeletal Ultrasound

**Figure 1:**
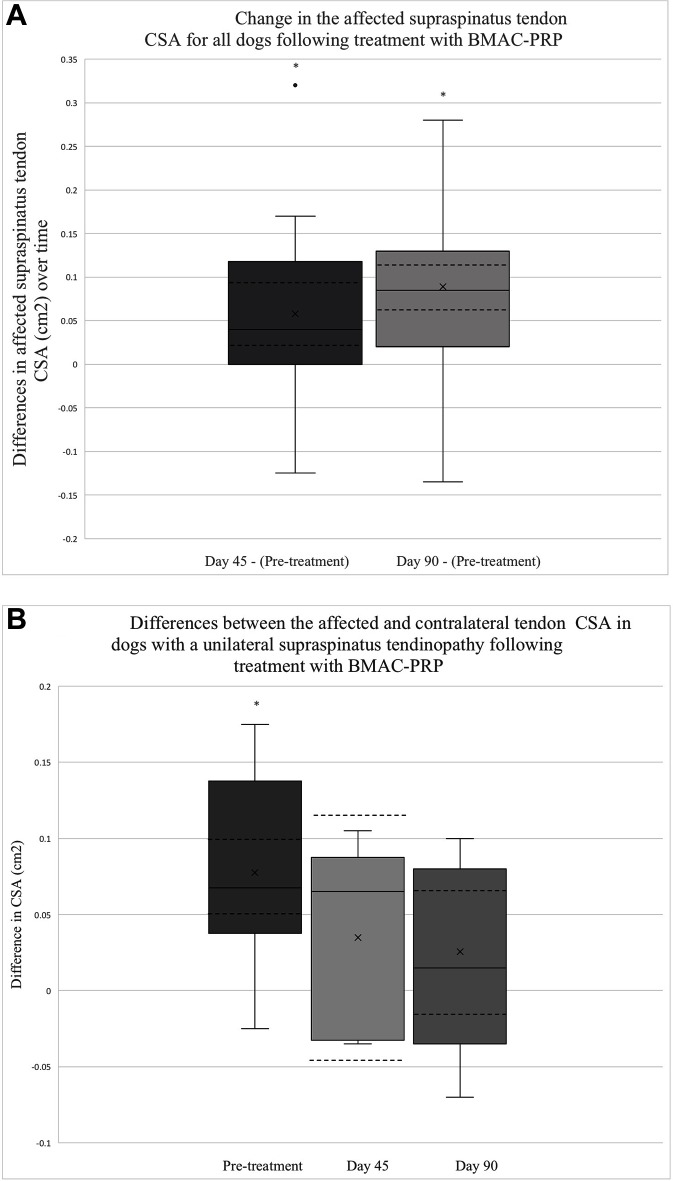
**(A) **This box plot depicts the distribution of change in the affected tendon CSA after treatment. The change in CSA between 45 days post-treatment and baseline (*N* = 26), as well as between 90 days post-treatment and baseline (*N* = 37) is depicted. The solid lines depict the median as well as the upper and lower quartiles. A single outlier is identified with a solid dot. The mean for each time point is identified by an x. The CI for each time point is also outlined by dashed lines. The asterisks indicate that the change in tendon CSA between the two time points are significant (Day 45 – Pre-treatment, *p* = 0.0025; Day 90 – Pre-treatment, *p* < 0.0001). **(B)** The distribution of differences between affected and contralateral limb tendon CSA prior to treatment (*N* = 20), 45 days post-treatment (*N* = 5), and 90 days post-treatment (*N* = 11) is displayed. The solid lines depict the median, upper, and lower quartiles. The mean for each point is identified by an x.The CI for each time point is also depicted using dashed lines. The asterisk highlights a time point at which the difference in CSA is significant (Pre-treatment – *p* < 0.0001, Day 45 – *p* = 0.2855, Day 90 – *p* = 0.1910).

Ultrasound CSA results were available for all 41 dogs at baseline, 26/41 dogs 45 days post-treatment and 37/41 dogs 90 days post-treatment. The mean CSA of the supraspinatus tendon for all dogs was noted to decrease significantly by 0.06 cm^2^ 45 days post-treatment (95% CI = 0.02,0.09; *p* = 0.0025) (Figure 1A). A larger decrease in CSA was observed between baseline and 90 days post-treatment at 0.09 cm^2^ (95% CI = 0.06,0.12; *p* < 0.0001) (Figure 1A).

Among dogs with a unilateral ST, a statistically significant difference was observed between the affected and unaffected CSA at baseline, with the affected tendon measuring an average of 0.08 cm^2^ larger than that of the unaffected tendon for the 20 patients in which this assessment was performed (95% CI 0.05,0.1; *p* < 0.0001) (Figure 1B). By 45 days post-treatment, this difference had decreased by 50%, with the affected tendon measuring only 0.04 cm^2^ larger than that of the unaffected tendon among the 5 patients in which this was assessed (95% CI −0.04,0.11; *p* = 0.2855) (Figure 1B). Ninety days post-treatment, the difference in CSA between the affected and unaffected limb was 0.03 cm^2^ among 11 dogs (95% CI −0.02,0.07; *p* = 0.1910) (Figure 1B).

**Figure 2:**
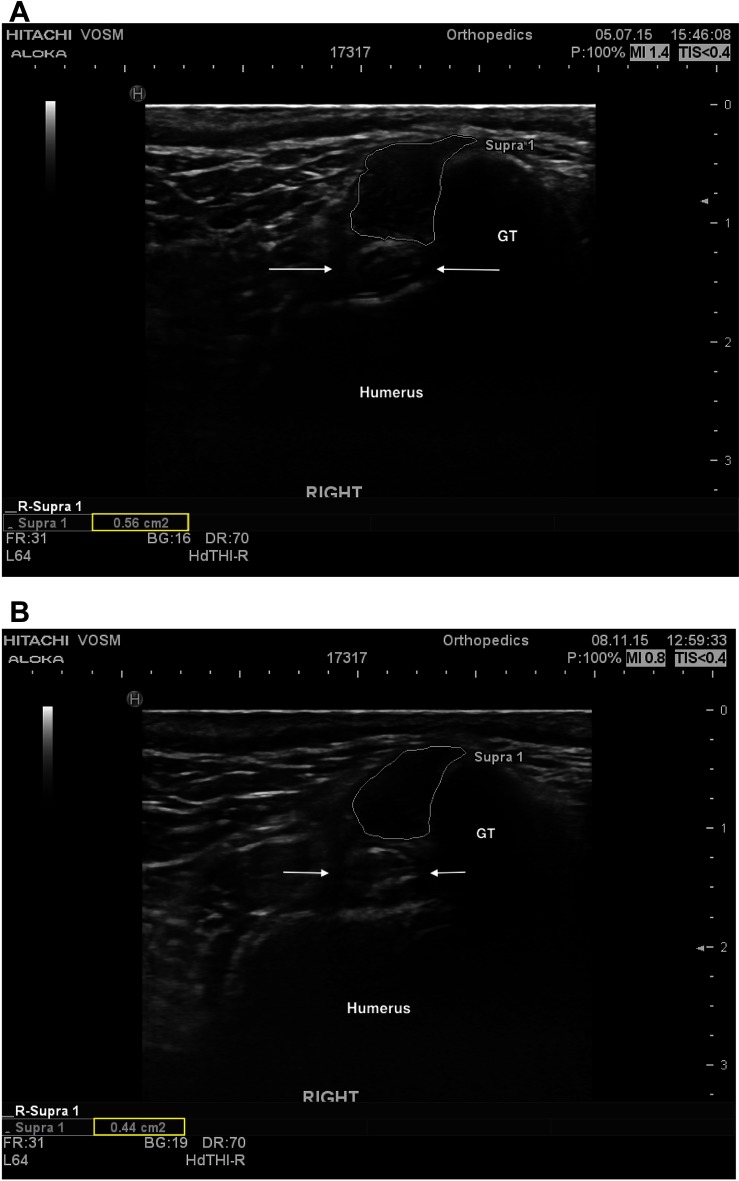
**(A)** A transverse ultrasonic view of the right supraspinatus tendon of patient A at the time of pre-treatment ultrasound. The supraspinatus tendon is highlighted (white outline), revealing a mottled heterogenous fiber pattern within the body of the tendon. No additional abnormalities were noted. For anatomical reference the greater tubercle (GT), humerus (humerus), and biceps tendon (arrows) are highlighted. **(B)** A transverse ultrasonographic view of the right supraspinatus tendon of patient A at the time of recheck ultrasound 96 days after intra-tendinous BMAC-PRP injection. The supraspinatus tendon is outlined. Note the homogenous, less echogenic (normal), fiber pattern within the body of the tendon.

**Figure 3:**
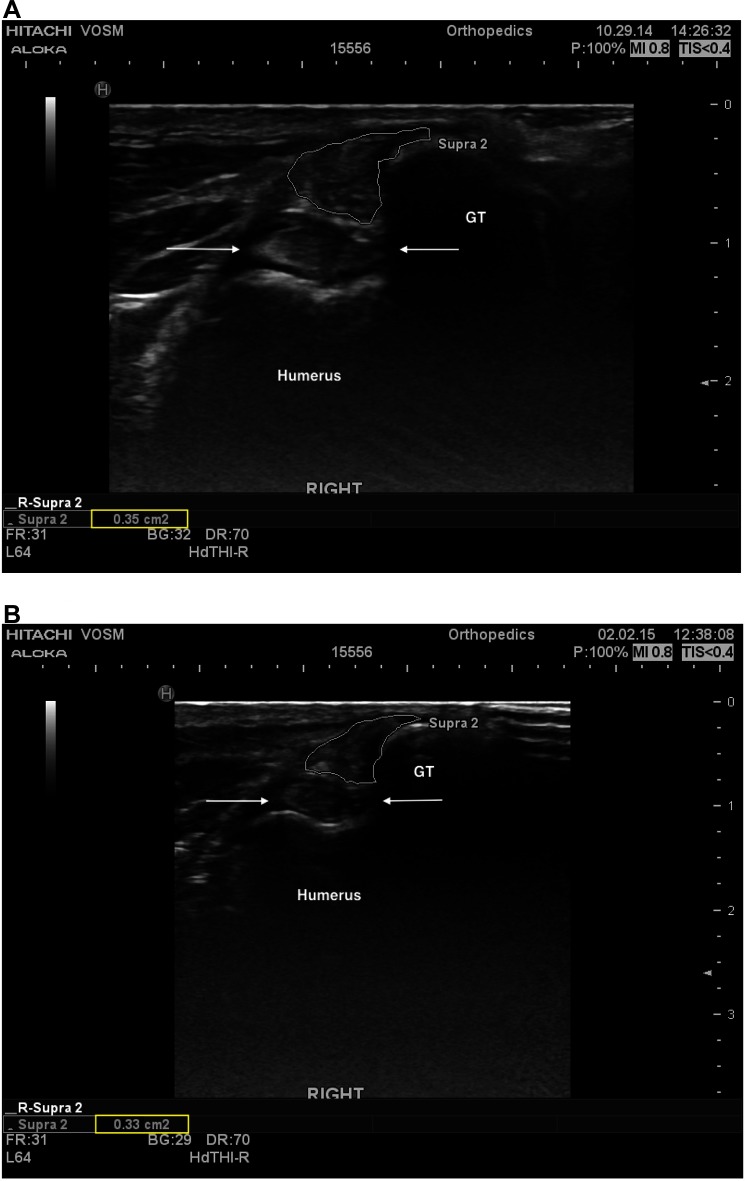
**(A)** A transverse ultrasonographic view of the right supraspinatus tendon of patient B at the time of pre-treatment ultrasound, prior to an intratendinous injection with BMAC-PRP. The supraspinatus tendon is outlined. Note the generalized mottled, hyperechoic, heterogenous fiber pattern. **(B)** A transverse ultrasonographic view of the right supraspinatus tendon of patient B at the time of a recheck ultrasound 96 days post treatment. The supraspinatus tendon is outlined. Note the static generalized mottled, heterogenous fiber pattern. Subjectively the sonographer noted that the baseline ultrasound revealed intratendinous inflammation that had largely resolved by this time, with primarily fibrous changes remaining.

Information regarding fiber pattern and echogenicity was available for 36/41 dogs at baseline and 32/41 dogs 90 days post-treatment. In most cases (29/32, 90.6%) the fiber pattern and echogenicity of the affected tendon was considered improved when a comparison was performed between baseline and 90 days post-treatment (Figure 2A,B). In the remaining cases (3/32, 9.4%) the fiber pattern and echogenicity was considered unchanged (Figure 3A,B). Though improved, the fiber pattern and echogenicity returned to normal in a minority of cases 4/32 (12.5%).

Specific sonographic findings for these dogs have been summarized in Table 1. The prevalence of most abnormalities were noted to decrease over time. A statistically significant decrease in the presence of a generalized hypoechoic fiber pattern (*P* = 0.003391) and contact/impingement of the biceps tendon (*P* =< 0.00001) was noted.

**Table 1:** Frequency and distribution of specific sonographic abnormalities in dogs with a supraspinatus tendinopathy pre-treatment and 90 days post-treatment with BMAC-PRP.

		Mottled, heterogenous fiber pattern	Generalized hypoechoic fiber pattern	Hypoechoic or anechoic foci	Hyperechoic foci	Evidence of calcification	Contact/Impingement of the biceps tendon
Pre-treatment	Absent	5 (14.3%)	20 (55.6%)	28 (77.8%)	9 (25.0%)	33 (91.6%)	7 (20.6%)
	Present	31 (85.7%)	16 (44.4%)	8 (22.2%)	27 (75.0%)	3 (8.3%)	27 (79.4%)
Day 90	Absent	7 (33.3%)	25 (89.3%)	24 (85.7%)	6 (19.4%)	24 (85.7%)	27 (84.4%)
	Present	14 (66.6%)	3 (10.7%)	4 (14.3%)	25 (80.6%)	4 (14.3%)	5 (15.6%)
p-value		*p* = 0.082389	***p* = 0.003391**	*p* = 0.419684	*p* = 0.580461	*p* = 0.449124	***p* < 0.00001**

Relationships between categorical data was assessed using the chi-squared or Fisher’s exact test. The resultant p-values can be seen at the bottom of each column. P-values are bolded where a relationship was detected between the groups at an alpha of 0.05.

## 4. Discussion

Similar to dogs treated with ADPC-PRP, dogs treated with BMAC-PRP demonstrated a decreased CSA in the affected supraspinatus tendon 90 days post-treatment (16). Additionally, in dogs that were unilaterally affected, the CSA of the enlarged supraspinatus tendon was found to return to that of the sonographically normal, contralateral tendon over the 90 day course of the study (16). This suggests that BMAC-PRP, like ADPC-PRP may be effective in reducing the size of the affected tendon in dogs diagnosed with an ST.

Impact on tendon size may be even more significant, given previous links between tendon size and tendon pathology. For example, tendon size is thought to increase sonographically in both acute and chronic tendinopathies due to etiopathalogic changes within the tendon (1). Acutely, hemorrhage and edema add to tendon bulk (1, 50). Chronically, the tendon likely remains thickened due to the presence of fibrous tissue, intratendinous calcification, and degenerative changes, including an increased proteoglycan content, loss of collagen organization and bundling, and myxomatous degeneration (48–60). 

As might be expected, in contrast with tendon injury, tendon healing is associated with a sonographic resolution of enlargement, along with a histologic increase in formation and organization of mature collagen and a decrease in cellularity, as demonstrated in Crass et al, a study investigating the correlation between sonographic and histologic changes that occur during equine tendon healing (60).

Though additional canine studies are indicated, these results suggest that a decrease in tendon size noted following treatment with BMAC-PRP may also be linked to a histologic improvement in tendon pathology.

In the current study, the changes in CSA were relatively small, with the largest mean change in CSA, 0.09 cm^2^, identified between baseline and 90 days post-treatment. When evaluated as a fraction of tendon size, however, this difference represented a 15.2% change in the size of the tendon. Though it was not within the scope of the current study to evaluate patient function alongside ultrasonography, similar changes have been considered significant in equine medicine, where a 10% increase in the size of the superficial digital flexor tendon is considered evidence of re-injury (21). Functional canine studies are needed to determine whether there is a similar correlation between the magnitude of change in tendon size and tendon function in dogs. Pending further evaluation, small changes in supraspinatus tendon CSA should be carefully considered in conjunction with clinical parameters.

In addition to changes in tendon CSA, diagnostic ultrasound also revealed improvements in echogenicity and fiber pattern in most (90.6%) cases. This is comparable to dogs receiving ADPC-PRP, 100% of which showed improvement in echogenicity and fiber pattern at follow-up (16). Notably, a greater proportion of dogs treated with BMAC-PRP and ADPC-PRP demonstrated sonographic improvement in fiber pattern and echogenicity than those treated with intratendinous PRP alone, where improvement was noted in only 9/16 (60%) patients 6 weeks after treatment (17). One likely contributor to this disparity, is the presence of stem cells in both BMAC and ADPC. These cells may differentiate into tenocytes, directly contributing to tendon regeneration (28). More importantly, however, MSCs exert indirect effects, including the release of cytokines and growth factors that recruit circulating stem cells to the site of injury, stimulate neovascularization, increase collagen density, and inhibit apoptosis and fibrosis, facilitating restoration of normal tendon architecture and biomechanical strength (24, 28–32).

Despite overwhelming improvement in fiber pattern and echogenicity, only a minority (13.8%) of cases were reported to have a normal fiber pattern and echogenicity 90 days post-treatment. This lack of complete response may be related to the brevity of follow-up with respect to tendon healing, a process that is highly variable, but generally takes approximately 3–5 months for sonographic resolution of architectural abnormalities (1, 61). In the current study, standard hospital recommendations for patient re-evaluation following the administration of BMAC-PRP limited the number and timing of post-treatment evaluations available for data collection. Future studies should aim to continue data collection for at least 5 months post-treatment to evaluate continued improvements in tendon fiber pattern and echogenicity.

Alternatively, it has also been proposed that almost all tendon injuries will be associated with chronic, minor ultrasound changes (1). This suggests that determining whether abnormalities are present, rather than evaluating each ultrasound for improvement, may result in an incorrect diagnosis of a persistent ST for some dogs. Diagnostics with a higher specificity may include an evaluation of improvement in tendon abnormalities over time, evaluation of the presence or absence of specific abnormalities over time, as previously mentioned, comparison to the contralateral limb in patients where a bilateral ST is not diagnosed, or the creation of a sonographic grading system. Paired functional studies are required to determine, which of these diagnostics would be clinically useful, if any.

Hypoechogenicity is related to intratendinous edema, which is commonly associated with acute tendinopathies, though it may also play a role in chronic tendinopathies following a failure of adequate remodeling (1, 10). Secondary to the enlargement that accompanies tendon edema, the supraspinatus tendon may contact the biceps. In more severe cases, this may progress to supraspinatus impingement of the biceps tendon. As both hypoechogenicity and contact of the biceps tendon are associated with tendon inflammation, the resolution of these findings following treatment with BMAC-PRP, suggests that there may be an association between treatment and resolution of intratendinous edema.

In addition to improvements in echogenicity and contact with the biceps tendon, a near-significant improvement was also noted in the prevalence of a mottled or heterogenous fiber pattern (*p* = 0.08239), commonly associated with fiber disruption, an acute partial tear or intratendinous hemorrhage (1). As with those changes noted above, improvement in this finding may suggest a resolution of acute pathology following treatment with BMAC-PRP. This might be expected, given the potent anti-inflammatory and immunomodulatory qualities of mesenchymal stem cells and PRP discussed above (24, 28–32). The small sample size in the current study may have resulted in a lack of statistical significance.

In contrast with the ultrasound findings above, evidence of intratendinous calcification was noted to increase, though the change was not statistically significant. In musculoskeletal sonography, acoustic shadowing is indicative of calcification, a chronic tendinous change (1, 23, 62). Therefore, it is possible that this increase is related to a lack of BMAC-PRP treatment efficacy with respect to intratendinous calcification. This is thought to be unlikely, however, as the presence of hyperechoic foci, also indicative of intratendinous calcification, (1) was noted to decrease following treatment. Alternatively, it is possible that a longer follow-up period is required to achieve resolution of tendon calcification. Lastly, it is important to note that calcification has been noted in dogs with no clinical signs of lameness (23, 62). As a result, signs of intratendinous calcification should be carefully interpreted, and be considered in conjunction with the patient’s clinical signs. In the current study, no functional data was collected, making interpretation of these findings difficult.

### 4.1. Limitations

The limitations of this study include those associated with retrospective data collection. Beyond the retrospective nature of this study, loss to follow-up also impacted our final cohort, reducing it by up to 21% for some outcome measures. This diminished the statistical power of the study.

Beyond the retrospective nature of this study, there was no attempt to blind the primary investigator (RM) to patient outcomes. As the study included no control group and the data had been recorded prior to data abstraction, this is not believed to have significantly biased the study results.

As a retrospective study, histopathologic assessment of the supraspinatus tendon was not possible. Histologic evaluation of the supraspinatus tendon would provide essential information regarding the impact of the administered stem cells, as well as their ability to differentiate and incorporate into the damaged tendon.

In the current study, dogs with concurrent orthopedic disease were not excluded, and data regarding additional orthopedic diagnoses was not collected. Given the high rate of concurrent shoulder and/or elbow pathology in dogs with an ST, (10) it is possible that the presence of additional orthopedic diseases in some dogs acted as a confounder. To this end, in a previous study investigating the use of ADPC-PRP for the treatment of STs, dogs with concurrent orthopedic pathology were excluded (16). When compared with these previous results, however, the ultrasound results obtained in the current study demonstrate similar trends and significant findings; therefore it is considered unlikely that the presence of concurrent orthopedic disease acted as a significant confounder for the above ultrasound findings.

Ideally, information regarding fiber pattern and echogenicity would have been evaluated quantitatively, allowing inter and intra-observer comparisons to be made, as well as objective evaluations of patients over time. For example, in human medicine, the ultrasound shoulder pathology rating scale (USPRS) is a quantitative scale that allows for an objective evaluation of the bony and tendinous components of the shoulder commonly involved in shoulder pathology, including the supraspinatus tendon (63). Unfortunately, no similarly validated scale has been published in veterinary medicine. Moreover, the supraspinatus component of the USPRS does not reflect the changes in fiber pattern and echogenicity noted in canine ST. For example, the USPRS does not evaluate calcification of the supraspinatus tendon or the type of change in echogenicity (i.e., hyper or hypoechogenic), both of which may suggest etiology, chronicity, and ultimately guide expectations following treatment (1, 3, 10, 23). To this end, information regarding fiber pattern and echogenicity was depicted in descriptive terms, rather than a numerical scale.

Although it is not considered a limitation, it was not within the scope of the current study to evaluate functional patient outcomes, such as measures of lameness over time. More importantly, though the presence of concurrent orthopedic disease was considered unlikely to confound sonographic findings of the supraspinatus tendon, the same could not be said for measures of lameness. Due to this inability to accurately isolate the impact of an ST on multifactorial lameness, evaluation of lameness was not performed in the current study. This would have provided an important correlate to the sonographic data collected in the current study.

Ultimately, additional, prospective ultrasound studies investigating the use of BMAC-PRP for the treatment of STs in dogs are necessary to determine how treatment efficacy compares with no treatment (placebo) and alternative treatments including NSAIDs, formal rehabilitation, and surgery.

To the author’s knowledge, only one previous study exists investigating the use of BMAC-PRP in the treatment of a tendinopathy (64). In this study, BMAC-PRP was delivered to humans with rotator cuff pathology, concluding that BMAC-PRP enhanced the proliferation and migration of tendon-derived stem cells, and suppressed inappropriate chondrogenic and osteogenic differentiation of these cells (64). A decrease in sonographic measurement of rotator cuff tears was also noted, though the change was not significant (64). Overall these results are promising and build on previous *in vitro* knowledge of the benefits that BMAC-PRP may have on a naturally-occurring tendinopathies, supporting further clinical investigation of this regenerative combination.

### 4.2. Conclusion

Overall, treatment of an ST with BMAC-PRP was associated with a decrease in individual tendon CSA over time, and a return of the affected tendon CSA to that of the contralateral limb, where an unaffected contralateral tendon was available for evaluation. An evaluation of fiber pattern and echogenicity revealed an improvement in most cases. Resolution of architectural changes was rarely achieved, however, the follow-up period was short, and resolution of sonographic abnormalities may not be a realistic goal in a setting of chronic tendinopathy. Additional, prospective, controlled, studies are required to evaluate the clinical and functional benefits of BMAC-PRP, however, it appears to be a potentially viable treatment alternative for ADPC-PRP in dogs with an ST.

## Author Contributions

RM was responsible for data collection, analysis, and primary authorship. SC is the owner of the surgical practice where patients were diagnosed and frequently diagnosed patients, referring them for musculoskeletal ultrasound to be performed by DC. SC and DC were responsible for assisting RM and reviewing and editing the paper.

## Conflict of Interest Statement

The authors declare that the research was conducted in the absence of any commercial or financial relationships that could be construed as a potential conflict of interest.

## Footnotes

1 BMC Stem Cell Kit: Companion Regenerative Therapies, Newark, DE, USA

2 2 Executive Series Centrifuge II: Emcyte Corporation®, Fort Meyers, FL, USA

3 PurePRP® Kit: Companion Regenerative Therapies, Newark, DE, USA

4 L64 Linear probe/transducer : Hitachi Healthcare, Twinsburg, OH, USA
